# Effects of hepatitis B virus infection and strategies for preventing mother-to-child transmission on maternal and fetal T-cell immunity

**DOI:** 10.3389/fimmu.2023.1122048

**Published:** 2023-02-16

**Authors:** Huihui Lu, Weihua Cao, Luxue Zhang, Liu Yang, Xiaoyue Bi, Yanjie Lin, Wen Deng, Tingting Jiang, Fangfang Sun, Zhan Zeng, Yao Lu, Lu Zhang, Ruyu Liu, Yuanjiao Gao, Shuling Wu, Hongxiao Hao, Xiaoxue Chen, Leiping Hu, Mengjiao Xu, Qiqiu Xiong, Jianping Dong, Rui Song, Minghui Li, Yao Xie

**Affiliations:** ^1^ Department of Hepatology Division 2, Beijing Ditan Hospital, Capital Medical University, Beijing, China; ^2^ Department of Obstetrics and Gynecology, Wuhan Children’s Hospital (Wuhan Maternal and Child Healthcare Hospital), Tongji Medical College, Huazhong University of Science and Technology, Wuhan, China; ^3^ Department of Infectious Diseases, Miyun Teaching Hospital, Capital Medical University, Beijing, China; ^4^ Infectious Disease Department, Xuanwu Hospital, Capital Medical University, Beijing, China; ^5^ Department of Hepatology Division 2, Peking University Ditan Teaching Hospital, Beijing, China; ^6^ Department of General Surgery, Beijing Ditan Hospital, Capital Medical University, Beijing, China; ^7^ Department of Infectious Disease, Haidian Hospital, Beijing Haidian Section of Peking University Third Hospital, Beijing, China; ^8^ Department of Infectious Disease, Beijing Ditan Hospital, Capital Medical University, Beijing, China

**Keywords:** hepatitis B virus, effects, pregnancy, mother-to-child transmission, immunological characteristics, T-cell immunity

## Abstract

One of the most common routes of chronic hepatitis B virus (HBV) infection is mother-to-child transmission (MTCT). Approximately 6.4 million children under the age of five have chronic HBV infections worldwide. HBV DNA high level, HBeAg positivity, placental barrier failure, and immaturity of the fetal immune are the possible causes of chronic HBV infection. The passive-active immune program for children, which consists of the hepatitis B vaccine and hepatitis B immunoglobulin, and antiviral therapy for pregnant women who have a high HBV DNA load (greater than 2 × 10^5^ IU/ml), are currently two of the most important ways to prevent the transmission of HBV from mother to child. Unfortunately, some infants still have chronic HBV infections. Some studies have also found that some supplementation during pregnancy can increase cytokine levels and then affect the level of HBsAb in infants. For example, IL-4 can mediate the beneficial effect on infants’ HBsAb levels when maternal folic acid supplementation. In addition, new research has indicated that HBV infection in the mother may also be linked to unfavorable outcomes such as gestational diabetes mellitus, intrahepatic cholestasis of pregnancy, and premature rupture of membranes. The changes in the immune environment during pregnancy and the hepatotropic nature of HBV may be the main reasons for the adverse maternal outcomes. It is interesting to note that after delivery, the women who had a chronic HBV infection may spontaneously achieve HBeAg seroconversion and HBsAg seroclearance. The maternal and fetal T-cell immunity in HBV infection is important because adaptive immune responses, especially virus-specific CD8 T-cell responses, are largely responsible for viral clearance and disease pathogenesis during HBV infection. Meanwhile, HBV humoral and T-cell responses are important for the durability of protection after fetal vaccination. This article reviews the literature on immunological characteristics of chronic HBV-infected patients during pregnancy and postpartum, blocking mother-to-child transmissions and related immune mechanisms, hoping to provide new insights for the prevention of HBV MTCT and antiviral intervention during pregnancy and postpartum.

## 1 Introduction

The presence of the hepatitis B virus (HBV) in the body for a period of more than 6 months is considered to be a chronic HBV infection. Chronic HBV infection is the primary factor that leads to liver cirrhosis and hepatocellular carcinoma (HCC). In 2019, there were roughly 316 million persons in the world who were living with chronic HBV infection, and the chronic HBV infection rate was 4.1% across the globe. The number of deaths that can be attributed to HBV rose by 5.9% between the years 1990 and 2019, and it rose by 2.9% between 2015 and 2019 (1). In countries with a high prevalence rate of chronic HBV infection, the most prevalent form of chronic HBV infection is mother-to-child transmission (MTCT). About 75 million women of childbearing age worldwide are infected with chronic HBV, accounting for about 25.3% (2, 3). In the absence of HBV preventive measures, the MTCT incidence of HBV infection is 31.3%; in addition, about 95% of cases infected during infancy or early childhood almost always result in chronic hepatitis (2, 3). Therefore, preventing HBV MTCT and the success of preventative measures taken for neonates are of great importance to human health.

Several guidelines recommend that pregnant women with a high level of HBV DNA should take anti-HBV drugs (like tenofovir disoproxil fumarate (TDF)) during the second or third trimester (4–7) to decrease the amount of HBV DNA in the mother, which in turn will lower the chance of HBV MTCT. The guidelines for HBV-infected pregnant women during pregnancy and postpartum are shown in **Table 1**. After birth, children should take preventive measures, namely regular injections of the hepatitis B vaccine and hepatitis B immunoglobulin (HBIG). The use of antiviral drugs during pregnancy and the injection of the hepatitis B vaccine and HBIG into the newborn greatly reduce the failure rate of HBV MTCT. In children younger than 5 years of age, in 2019, the hepatitis B surface antigen (HBsAg) prevalence rate was 1% across the world, which was 76.8% lower than the prevalence rate in 1990 (1). However, there are still some newborns that are afflicted with HBV, particularly those born to pregnant women who either have a high HBV DNA load or are positive for the hepatitis B e antigen (HBeAg) (4, 8). Therefore, some studies pointed out that the implementation of strategies for blocking MTCT of HBV should not only consider HBV DNA load but also combine HBeAg status to achieve a higher degree of precision in determining the risk of HBV MTCT. In addition, the HBeAg test can be used as an indicator to determine whether or not antiviral treatment is required during pregnancy in regions where the HBV DNA test is not available (9). In 2016, the World Health Organization (WHO) made a proposal to reduce the number of new cases of hepatitis by 90% in the year 2030 (10). Therefore, aggressive and safe antiviral and immunological trials based on pregnant women are required to lower the risk of HBV-related unfavorable consequences for both the mother and the offspring.

**Table 1:** Guidelines for HBV-infected pregnant women during pregnancy and postpartum.

Guideline	Year of publication	Pregnant women who should receive antiviral intervention	Start antiviral intervention gestational week	First-choice antiviral drug	Puerpera when should discontinue the antiviral intervention	Postpartum monitoring
European Association for the Study of the Liver (EASL) (7)	2017	HBV DNA >200,000 IU/ml or HBsAg >10,000 IU/ml	24–28 weeks	TDF	Continue for up to 12 weeks after delivery	–
American Association for the Study of Liver Diseases (AASLD) (4)	2018	HBV DNA >200,000 IU/ml	28–32 weeks	TDF	Immediately after delivery to 3 months postpartum	Postpartum 6 months for hepatitis flares and seroconversion
Chinese Society of Infectious Diseases, Chinese Medical Association (6)	2019	HBV DNA >200,000 IU/ml	24–28 weeks	TDF	Immediately after delivery or continue up to 1–3 months postpartum	Up to postpartum 6 months
Asian Pacific Association for the Study of Liver (APASL) (5)	2022	HBV DNA >200,000 IU/ml	24–28 weeks	TDF	Immediately after delivery or continue up to 12 weeks postpartum	At least 24 weeks for hepatitis fare and rebound of HBV DNA

## 2 Biological characteristics of HBV

**Figure 1:**
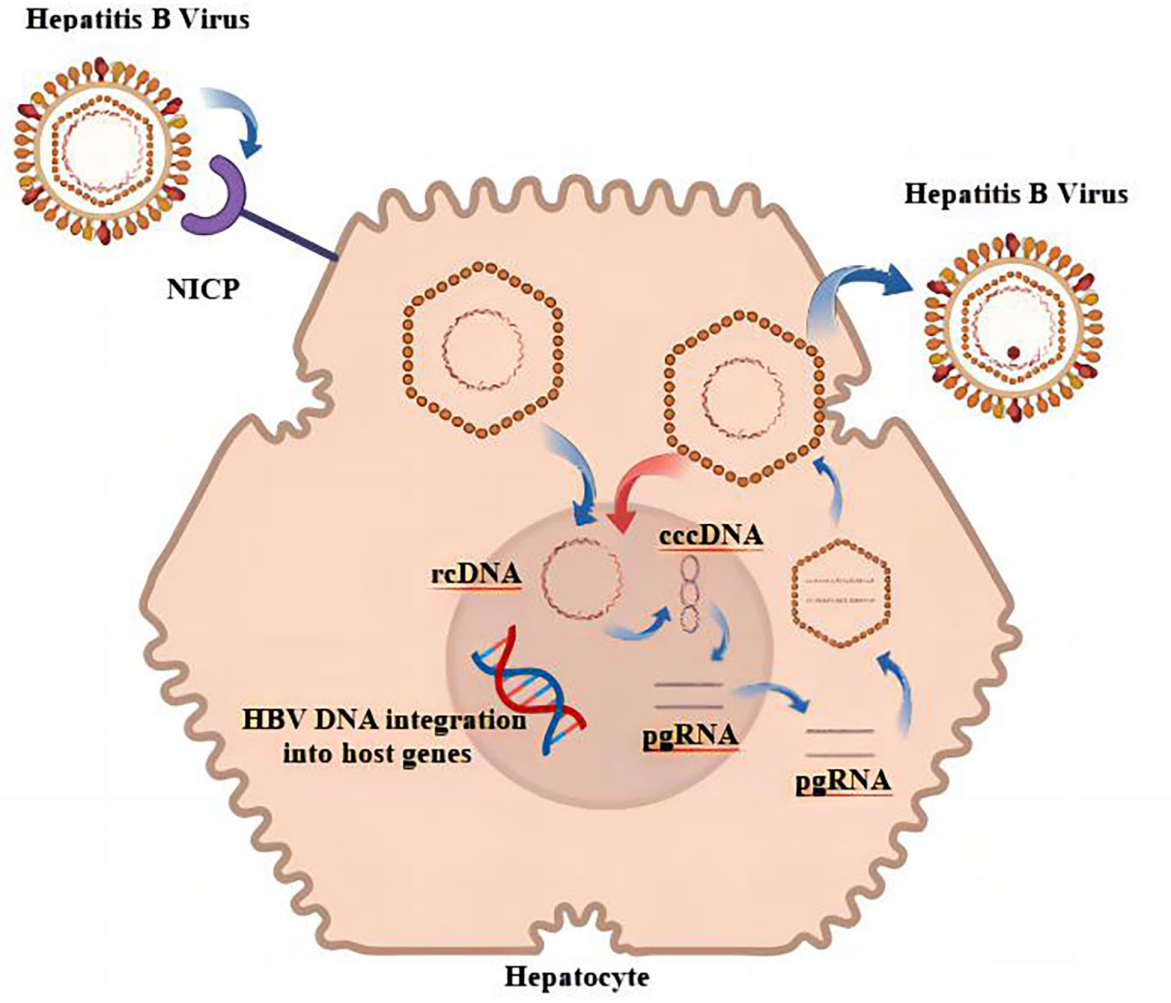
Replication cycle of HBV.

As a hepatotropic virus, HBV can be integrated into the genome of host hepatocytes after infecting humans, and the integrated HBV is a factor in the continuous generation of viral proteins as well as the induction of HCC (11, 12). HBV releases its genome into the host liver nucleus, where it establishes a DNA pool that is full of covalently closed circular DNA (cccDNA); the pool is characterized by persistence and stability (13). In addition, a number of studies came to the conclusion that the sources of HBsAg are different in various phases of chronic HBV infection and that cccDNA was a primary source of HBsAg in HBeAg-positive persons (14). When HBeAg was negative, it is possible that HBV DNA integration was the main source of HBsAg in HBV-infected individuals (15). It is also one of the reasons why it is difficult to achieve serological disappearance of HBsAg in CHB treatment. The replication cycle of HBV is shown in **Figure 1**. The likelihood of developing HCC would drop dramatically after HBsAg was no longer present (16). Unfortunately, this risk has not disappeared entirely. Studies had shown that patients who had achieved functional cures are still at the risk for HCC, which might be related to the influence of continuous HBV integration on the promotion of HCC (17). HBV integration plays a similar biological function in promoting HCC, regardless of whether HBsAg is positive or negative (18). The biological characteristics of HBV that have been discussed imply that one of the essential steps toward decreasing the number of worldwide HBV-related adverse events is the complete and total elimination of HBV MTCT, as well as the reduction in the number of new cases.

## 3 Adverse maternal outcomes and HBV infection

Whether HBV infection can affect maternal immunity and lead to adverse maternal outcomes is still controversial. According to the findings of a retrospective cohort study, having a positive HBsAg was associated with an increased risk of a number of adverse maternal outcomes during pregnancy; these adverse maternal outcomes included gestational diabetes mellitus (GDM), postpartum hemorrhage (PPH), cesarean delivery, and intrahepatic cholestasis (19). Zhang et al. (20) retrospectively analyzed the pregnancy outcomes of 9,699 HBsAg-positive and 73,076 HBsAg-negative pregnant women, and the result found that pregnant women who were HBsAg-positive were more likely to undergo PPH and intrahepatic cholestasis of pregnancy; in addition, HBsAg-positive pregnant women had a higher risk of placental abruption and premature birth in the vaginal delivery group. According to the findings of another meta-analysis, the risk of preterm birth in pregnant women who had a chronic HBV infection was significantly higher than the risk of preterm birth in pregnant women who did not have an HBV infection; furthermore, the risk of preterm delivery rose by 16% (21). Moreover, the presence of HBV was an independent risk factor for early preterm delivery, but the level of HBV DNA did not affect the increase in risk associated with early preterm delivery (22). Peng et al. (23) demonstrated that baseline HBeAg status and HBV DNA level were not associated with GDM, despite the fact that maternal carrying of HBsAg was an independent risk factor for GDM. The occurrence of GDM may be related to age (> 35 years old) and abnormal liver function (24). However, advanced age is a risk factor for GDM (25). According to the findings of another study, chronic HBV infection was linked to a marginally increased risk of low birth weight and preterm birth, but HBeAg positivity led to an increased risk of low birth weight, preterm birth, and GDM (26).

According to some studies, there was no difference in the risk of preterm delivery or GDM between the HBsAg-positive group and the non-HBV-infected group; however, the abortion rate of the non-HBV-infected group was significantly less than that of the HBsAg-positive group before or after using the multivariate model to adjust the confounding factors (sociodemographic variables and obstetric complications) (27). Bajema et al. (28) believed that there was no correlation between having a positive HBV and an increased risk of adverse pregnancy outcomes in a country that had a low epidemic situation (the USA). In addition to the adverse outcomes of preterm birth, GDM, and abortion, alanine aminotransferase (ALT) flare in HBV-infected women during pregnancy or postpartum is also of great concern for the reason that the maternal immunity may change after delivery (29, 30). A prospective study involving 158 chronic HBV-infected pregnant women showed that during pregnancy or postpartum, spontaneous elevation of ALT in HBV-infected women was infrequent, mild, and self-limited; after birth, the group of women discontinuing antiviral medication was more likely to have ALT flare (AF, defined as ≥100 U/L), and the AF was also self-limiting and mild (31). Another study of untreated women with HBV infection during pregnancy and postpartum, which excluded women who started antiviral therapy during pregnancy for postnatal analysis, found a sudden increase in ALT and HBV DNA during the third trimester and early postpartum period in women with CHB; however, baseline ALT, HBV DNA, HBeAg-positive status, gestational age, and delivery times were not predictors of undesirable clinical outcomes, including spontaneous abortion, hepatic flare, premature delivery, GDM, pre-eclampsia, and liver failure (32). Another Chinese study involving 869 HBV-infected patients who did not receive antiviral intervention and 3,367 pregnant women without HBV infection showed that elevated ALT levels were more common in the CHB group than those in the HBV-free group; women with HBV DNA levels ≥ 5 log10 IU/ml or increased ALT during delivery are more likely to have a sudden increase or worsening of ALT (33). Therefore, compared with other adverse consequences, elevated ALT after delivery is common in pregnant women with HBV infection, and they should be followed up closely after delivery regardless of whether they have taken antiviral drugs during pregnancy. Mild elevations in ALT are usually self-limiting. If the ALT flares (≥ five times ULN), it should be combined with other viral markers (such as HBV DNA lever and HBeAg state) to determine the need for antiviral therapy. With antiviral therapy, ALT can usually be controlled and rarely leads to serious consequences such as liver failure.

## 4 T cells and HBV infection

### 4.1 Maternal T-cell immunity and HBV infection

HBV infection affects maternal immunity, resulting in adverse maternal outcomes that may be related to pregnancy and T-cell mechanisms. T-cell-induced immune mechanisms also play an important role in the phenomenon of allograft tolerance zygote formation, embryo implantation and development, and fetal allograft tolerance. The fetus is a combination of chromosomes of both parents, and for the mother, the zygote is an allograft. The success of pregnancy is bound to be accompanied by the successful suppression of immune rejection and the establishment of maternal immune tolerance, and the dynamic balance of immune cells controls the whole process from the successful implantation of the embryo to the delivery of the fetus (34). The inhibition of Th1 response and activation of Th2 immunity would impair maternal immune reaction to HBV and reduce CD8 T cells; then, the impaired immune led to virus activation and immune escape, resulting in increased vertical transmission for the reason that CD8 T cells were the main effector cells in T-cell response (35). Progesterone has the ability to prevent threatened abortion (36). Th2-related cytokines such as interleukin-4 (IL-4) and interleukin-10 (IL-10) can promote progesterone production by luteal cells, and the promoting effect is more significant in luteal cells in early pregnancy (37). The study by Piccinni et al. also found a significant increase in decidual T cells and the contents of leukemia inhibitory factor (LIF), IL-4, and IL-10 in women who were normal gestation compared with decidual cells in women with recurrent miscarriage (38). At the fetal-maternal interface, IL-4 and LIF mediated by progesterone contributed to the success and maintenance of pregnancy. Furthermore, LIF expression by T cells was positively correlated with IL-4 production, implying that progesterone could promote LIF expression by T cells by inducing the production of IL-4, which could be one of the mechanisms by which progesterone worked to maintain normal pregnancy (38). According to the findings of another study, both IL-4 and IL-10 could suppress the maturation and function of Th1 cells as well as macrophages, which helped to prevent allograft rejection (39). The changes in decidual memory T-cell subsets and related cytokines may also be related to the occurrence of pre-eclampsia (40). Chronic HBV infection changed the immune response of women with reproductive failure by increasing the frequency of B cells, decreasing the number of CD3^+^CD4^+^ helper T cells, and reducing the activity and toxicity of peripheral NK cells (41). Although there is no clear conclusion on the adverse influences of HBV infection on mother and fetus in different studies, in the era of precision medicine, it is necessary to closely detect HBsAg-positive pregnant women during pregnancy and postpartum. As an immune-involved disease, T-cell immunity plays an important role in the elimination of HBV. The adverse events induced by HBV infection may be because HBV disrupts the maternal–fetal immune balance by interfering with the activation of T cells or the secretion of related cytokines, resulting in a series of pregnancy and postpartum complications. In contrast, it is possible that HBV infection in those uninfluenced by HBV infection is in equilibrium with T-cell immunity.

Gao et al. (42) analyzed the immune microenvironment of pregnant women in their second trimester (22–25 weeks), who were either healthy pregnant women without HBV infection or pregnant women with a positive state of HBeAg and HBV DNA ≥ 1.0 × 10^7^ IU/ml; the results by analyzing T-cell receptor sequencing in mononuclear cells taken from peripheral blood showed a more active immune response among the immune cell subsets in HBV-related samples; more specifically, HBV-infected pregnant women had a higher percentage of effector/memory CD8^+^ T cells, and both CD4^+^ and CD8^+^ effector/memory T cells of HBV-infected pregnant women increased expression of genes related to metallothionein at higher levels, activated more metal ion pathways, and caused a variety of inflammatory responses. Another interesting thing was that even women with a normal pregnancy (non-HBV infection) also had immune effects, which might last until approximately 1 year after delivery. During early pregnancy, for instance, there is a rise in suppressor T cells that help the mother accept the fetus and a rise in helper T cells that aid in pregnancy maintenance; furthermore, the postpartum aggravation of autoimmune diseases may be associated with the activation of helper T cells and cytotoxic T cells between 1 and 4 months postpartum and the activation of suppressor T cells between 7 and 12 months postpartum (39). The changes of pregnancy to the immune system, particularly T-cell-associated immunity, can lead to elevated ALT levels during pregnancy or the postpartum period in women who have a chronic infection with HBV.

### 4.2 Fetal immunity and HBV infection

#### 4.2.1 Placental immunity and viral infection

As a link between mother and fetus, the placenta has the functions of material exchange, barrier, immunity, and others (43). For some substances, however, the placental barrier does not completely block MTCT. In the case of viral infections, some viruses have developed innate immune mechanisms to evade the placental barrier. The most common “TORCH” testing includes Toxoplasma gondii, other viruses (such as *Treponema pallidum*, parvovirus B19, varicella-zoster virus), herpes simplex virus 1 and 2, rubella, and cytomegalovirus (44). These viruses might cause a series of adverse events, such as early abortion, fetal organ malformations (such as congenital heart disease (45)), functional defects of organs (such as sensorineural hearing loss in children (46)), and so on. Unfortunately, some defects in fetal organ function are not detected until after childbirth, for the reason that prenatal testing (such as ultrasound during pregnancy) is unable to identify them (such as amentia) before delivery. Therefore, the TORCH examination is one of the recommended examinations for women who are ready for pregnancy or who are in early pregnancy. In addition to those effects during pregnancy and the perinatal period, viral infections can also make babies more susceptible to a number of diseases. Some scholars believed that this observation was related to the impact that early-life virus infection has on the immune system. In their studies, they found that infants infected with cytomegalovirus within the first year of life had a higher risk of tuberculosis in childhood (47). Similar to the above viruses, HBV or HBV-related markers can cross the placental barrier to cause intrauterine infection, which is one of the possible mechanisms of HBV intrauterine infection, which ultimately leads to the failure of MTCT treatment for HBV and undesirable outcomes. Failure to block MTCT of HBV is usually associated with HBeAg positivity and/or high HBV DNA levels, which are important virological factors, and the intrauterine infection rate of children born to such mothers is 1%–9% (48). In addition, infants who were younger with gestational age at birth or who have not received three complete doses of the hepatitis B vaccine were also prone to failure of MTCT of HBV (49).

#### 4.2.2 Placental immunity and HBV markers

Intrauterine transmission (IUT) is one of the ways of MTCT of HBV. At present, little is known about the clear mechanism of IUT caused by HBV. There are many factors that lead to IUT with HBV. In addition to the positive status of HBeAg and/or high levels of HBV DNA, these negative factors also include the poor immune status of the mother, the presence of minor thalassemia, HBV mutation, disruption of placental barriers, and fetal susceptibility (50–53). Some research on the mechanism of intrauterine HBV infection has shown that the levels of HBV or its related markers decrease from the maternal surface to the fetal surface of the placenta. However, whether HBV or its related markers replicate in the placenta remains controversial. Some scholars have studied the derivatives of HBV. Derivatives such as HBsAg-positive and HBV DNA-positive were markers of HBV infection. When HBsAg is negative, HBeAg or HbcAb is important evidence of contact with HBV. Zhang et al. (54) found that both HBsAg and HBcAg can be found in placentas that come from mothers who were positive for HBsAg using ABC immunohistochemical method. They also detected that the level of two antigens decreased from the maternal side of the placenta to the fetus’ side. It also indicated that the placenta might act as a partial barrier to HBV. *In vitro* experiments, HBV translocated across a trophoblastic barrier by transcytosis of infectious hepadnavirus and HBV DNA, which were detected in placentas after the co-culture of HBV DNA-positive serum and trophoblast cells (55, 56). Garima et al. (57) also observed that even at relatively low levels, receptors and HBV markers could replicate in the placenta. As the placenta developed, trophoblast cells differentiated and fused into multinucleated cells that formed the syncytiotrophoblast, and the maturity of the placenta and specific maternal antibodies, such as maternal immunoglobulin G (IgG), had the ability to reduce the risk of HBV transmission (55). The placenta usually matures with gestational age, but in premature infants, the placenta may not mature due to gestational age or other factors (58). This could explain why premature babies are more likely to succumb to MTCT.

#### 4.2.3 Fetal immunity and HBV markers

HBeAg and HBsAg are two significant secretory proteins of HBV and have the function of affecting host immunity to maintain persistent HBV infection. The point had been confirmed in many studies. Another thing to consider is that maternal-derived HBeAg can spread throughout the placenta and be detected in newborns. In addition, such HBeAg altered macrophage function in infants and induced immunologic tolerance *in utero* to drive the persistence of HBV infection after vertical transmission in a mouse model (59, 60). However, another study believed that the source of the HBeAg-positive mother did not lead to T-cell tolerance toward products related to the HBV-related gene, and this result was based on the research about a mice model born to HBeAg-transgenic mothers. Fortunately, if HBV MTCT is successful, HBeAg in an infant will not always be in the body and cannot be detected before the first 6 months of life; anti-HBe becomes undetectable before the age of 1 year (61).

The HBsAg exposure *in utero* has not been completely investigated to determine whether or how it affects the continued presence of HBV replication and the fetal immune response. Through the research on a mouse model of only HBsAg exposure *in utero*, Ning et al. came to the first conclusion that, contrary to what others had previously thought, HBeAg played an immunosuppressive effect on the offspring; only HBsAg exposure *in utero* did not develop the immune tolerance to HBV on the fetus; however, when re-exposed to HBV, the offspring could speed up the elimination of HBV in the body; and this beneficial effect may be related to only HBsAg exposure *in utero*, which could activate antigen-presenting dendritic cells and then induce an increase of the HB-specific CD8^+^ T cells and secretion of interferon (IFN)-γ in the hepar of mice (62). Another research by cord blood analysis also showed that immunity of newborn infants can be beneficially affected due to the HBV-infected state of their Asian mothers; it was mainly manifested as the enhancement of innate immune cell maturation and the increasement of Th1 development, and specially showed that low levels of IL-10 (Th2-related cytokines) and high levels of IL-12p40, IFN-α2 (Th1-related cytokines); importantly, such enhancement effect led to the robust response of the neonatal immune cells when they were exposed *in vitro* to pathogens that were unrelated to their condition, and the pathogens were *Pseudomonas aeruginosa*, *Salmonella typhimurium*, uropathogenic, *Escherichia coli*, *Acinetobacter baumanii*, and *Listeria monocytogenes*; however, this beneficial effect cannot be demonstrated in the baby Caucasian HBV-infected mother’s delivery, which was explained by different HBV genotypes; patients of Asian descent had HBV genotypes B/C, while patients of Caucasian descent had HBV genotype D (63). From the result of their important experiments, we can infer that compared to the offspring of HBeAg-positive females, those born to HBeAg-negative females are less adversely affected by HBV infection and may be beneficially affected. A number of researchers had also conducted research on how chronic HBV infection altered the immune response at the maternal interface of the placenta. A study supported enrolled chronic HBV-infected pregnant women (HBV group) and pregnant women without HBV infection as a healthy control group (HC group), and showed that hyperactivated decidual CD8^+^ T cells in the HBV group generated a lower level of IFN-γ than those in the HC group, indicating that the ability of hyperactivated decidual CD8^+^ T cells had been impaired in the HBV group; it concretely demonstrated that the activation molecules of CD69 and HLA-DR in the HBV group were low; they suggested that in order to stop the spread of HBV through MTCT, antiviral intervention in the HBV group was necessary and important (64).

A function of CD8^+^ T cells was found to be associated with persistent HBV infection in newborns, as was discovered in other studies that investigated the mechanism behind chronic HBV infection. A study from the USA showed that there was not a significant difference between the three groups when it came to the frequency of CD8 and CD4 T cells in peripheral blood and cord blood that was collected at birth; two groups (positive for both HBV DNA and HBsAg: group 1; negative for both HBV DNA and HBsAg: group 2) were born to HBsAg-positive mothers, and another group (HBV DNA and HBsAg negative: group 3) was born to healthy mothers; but in comparison to groups 2 and 3, it was found that CD8^+^ T cells of group 1 had a functional defect that was characterized by decreased IFN-c production and decreased CD107A expression. In addition, significant differences were found among the three groups with regard to the frequency of regulatory T cells expressing FoxP3 in the peripheral blood, and the frequency of regulatory T cells expressing FoxP3 in group 1 was the highest of all three groups (group 1: 63.79%, group 2: 8.12; group 3: 11.06%, *p* < 0.05). In other words, CD8^+^ T cells in newborns who tested positive for HBV DNA and HBsAg were dysfunctional. It was possible that the immune tolerance state of the adaptive immune system in neonates who had chronic HBV infection was related to the expansion of regulatory T cells and the impairment of the TCR signal in these infants (65). Another study also showed that HBV or HBV-related markers could be exposed to the fetus *in utero*; when the HBV-related markers had enough concentration, they could stimulate fetal/neonatal immune responses; after HBcAg stimulation, increased IFN production was observed in 30.4% of infants with both negative HBsAg and HBeAg, and the infants were born to HBsAg-positive mothers (66).

## 5 T cells and measures of HBV-MTCT prevention

Anti-HBV intervention for the appropriate population and timely and standardized injection of HBIG and hepatitis B vaccine after birth are important measures of HBV-MTCT at present. The safety of anti-HBV drugs and hepatitis B vaccine for mothers and offspring has been confirmed in many clinical studies (67–70). It is suggested that the hepatitis B vaccine should be given at the first 24 h, 1 month, and 6 months of neonatal life. Moreover, the WHO also recommended one dose of HBIG combined with the hepatitis B vaccine to improve the success of HBV-MTCT prevention within 24 h of birth. The effect of anti-HBV drugs on maternal and offspring immune systems is also one of the issues worth exploring.

### 5.1 Maternal immune and measures of HBV-MTCT prevention

#### 5.1.1 Maternal immune and antiviral intervention

Huang et al. (71) demonstrated that T-cell immunity got involved in AF after delivery, and their study showed that compared with pregnant women who did not use antiviral intervention (NAF group), pregnant women who received the antiviral intervention (AF group) during pregnancy had significantly lower HBsAg levels at 6–8 or 15–18 weeks after delivery. In addition, CD4^+^ T cells or CD8^+^ T in the AF group produced fewer anti-inflammatory cytokines, such as IL-10, or more proinflammatory cytokines (tumor necrosis factor-α (TNF-α), IFN-γ, IL-2, IL-21) before, during, and after antiviral therapy; furthermore, the proportion of IFN-γ to IL-10 was higher in the AF group than that in the NAF group during pregnancy or postpartum. Another study enrolled HBV-infected pregnant women in the immune-tolerant phase of NA intervention at 24 to 28 weeks of gestation. According to the ALT levels at 6–12 weeks after delivery, the pregnant women were divided into the postpartum hepatitis group (group 1) and nonhepatitis group (group 2), and the results showed that compared to baseline or group 2, group 1 after childbirth usually had relatively lower levels of HBsAg, HBeAg, and HBV DNA. In addition, ALT flare after childbirth in immune-tolerant HBV patients may be associated with alterations in immune system performance. The result also showed that CD8^+^ T cells included CD8^+^ effector memory T cells and effector T cells that were significantly activated after childbirth in group 1 (72). A study found that compared with the group not taking antiviral drugs, the postpartum NK cell count in the group receiving antiviral therapy during pregnancy increased significantly, and postpartum hepatitis may also be related to the change of NK cell frequency and immune damage caused by HBV infection (73). Li et al. (74) conducted a prospective study to investigate whether antiviral therapy and the timing of postpartum drug withdrawal affected the risk of developing postpartum hepatitis among pregnant women infected with HBV, and the study showed that the timing of drug withdrawal (immediately or ≥ 6 weeks postpartum) did not have any impact on the rate at which postpartum hepatitis occurred in CHB women. Meanwhile, within 12 weeks after delivery, hepatitis occurred in more than 90% of the women who did not take antiviral drugs and those who stopped antiviral drugs immediately after delivery, but drug withdrawal at 6 weeks postpartum and more than 6 weeks postpartum may delay the onset of postpartum hepatitis. Another study revealed that 45% of women who had a chronic HBV infection showed activity of liver disease after delivery, and liver disease activity was defined as ALT within 6 months postpartum ≥ three times the lowest ALT value during pregnancy. Compared with those not treated with lamivudine, pregnant women receiving lamivudine intervention during pregnancy had a higher rate of liver disease activity after delivery (75).

T cells play an important role in anti-HBV therapy. HBV produces and secretes a large number of related markers and depletes B cells and specific T cells, resulting in impaired cytokine activation and continuous expression of a variety of inhibitory receptors, which enable it to evade the host’s innate immune system and continue to replicate in hepatocytes. Le et al. (76) analyzed the immune cells of patients with chronic HBV infection and found that the number of HBS-specific T cells in patients with chronic HBV infection negatively increased with age, and no correlation was found between serum HBsAg levels and the phenotype or function of T cells or NK cells. A study had also found that the escape mechanism might be connected to the manipulation of cell metabolome by HBV (77). CHB is an immune response-mediated disease, and nucleo(t)ide analog (NA) or IFN is an effective and widely used therapy for CHB patients. IFN has a stronger ability to clear HBsAg than NAs, which may be related to the fact that IFN can significantly promote the expression of a variety of immune cells, including NK cells and plasmacytoid dendritic cells (pDCs) (78, 79). In addition, CD8^+^ T-cell-mediated immunity has the function of anti-HBV, and NK cells have been found to contribute to the induction of CD8^+^ T-cell-mediated immunity in animal models (80). Therefore, IFN induces the expression of a variety of immune cells and indirectly induces host T-cell immunity, which may be the key to achieving HBsAg seroconversion. In addition, dynamic changes in cytokines are observed when CHB patients receive antiviral therapy (81). CHB patients receiving IFN antiviral therapy can achieve HBeAg serological conversion when the frequency of inhibitory cells (such as FoxP3(+) Tregs) is reduced (82). Overexpression of some chemokine ligands can also accelerate HBV clearance by activating depleted T cells. Yan et al. (83) observed that overexpression of C–C motif chemokine ligand 19 (CCL19), a leukocyte chemoattractant, could rapidly eliminate HBV in primary mouse hepatocytes. Their research also suggested that overexpression of CCL19 could increase the number of CD8^+^ T cells, reduce the frequency of PD-1^+^ CD8^+^ T cells in the blood, and weaken the inhibition of hepatic antigen-presenting cells (APCs). *In vitro* models, it was found that the T-cell conjugation antibodies were used to drive T cells to adsorb specifically to tumor tissues expressing HBV envelope proteins, and then by activating the antiviral activity of T cells, it could eliminate HBV-infected hepatocytes and specifically kill tumor cells (84). In conclusion, anti-HBV therapy and the elimination of HBV-related tumors both rely heavily on the contributions of T cells.

#### 5.1.2 The effect of HBIG implementation during pregnancy

The results on the effectiveness of HBIG implementation during pregnancy for MTCT are conflicting, and so far, this treatment has not been adopted by other countries. A meta-analysis included 36 trials of pregnant women receiving HBIG for the prevention of MTCT of HBV infection during the third trimester of pregnancy; the result showed that adverse events of the HBIG in newborns had not increased and HBIG could reduce HBsAg-MTCT in newborns compared with no intervention, but the investigator believed that the quality of the evidence found in this review was very low, so the result could not prove that HBIG was beneficial in preventing MTCT of HBV (85). Another study using immunohistochemical staining of the placentas of HBsAg-positive pregnant women found that HBsAg positivity was mostly found in trophoblastic cells and villous mesenchymal cells, while HBIG deposition mostly distributed in Hofbauer cell areas. There was no colocalization of HBIG and HBsAg in the placenta, and HBV DNA, HBeAg, and HBcAb load in peripheral venous blood from mothers receiving prenatal HBIG treatment did not differ significantly from that of rejected HBIG mothers. As a result, HBIG had no effect on virus replication; however, HBIG had the potential to form an immune barrier in the placenta, blocking HBV transmission from mother to fetus (86). Overall, rigorously designed, randomized, and prospective clinical trials should be conducted to determine the benefits and drawbacks of using HBIG in the prevention of MTCT of HBV.

### 5.2 Fetal immune and measures of HBV-MTCT prevention

#### 5.2.1 Fetal immune and antiviral intervention

Little information is available on the influence of antiviral drugs on neonatal immunity. A study from China showed that the mother used LDT for the prevention of HBV-MTCT, and in this condition, LDT had effect on CD4^+^CD25^+^ regulatory T cells of neonate. The researchers enrolled HBsAg-positive pregnant women who were divided into HBeAg-negative group (viral load < 10^6^ IU/ml) and HBeAg-positive group (viral load ≥ 10^6^ IU/ml). According to the willingness to receive LDT intervention, HBeAg-positive group was divided into the LdT-treated group and the nonntreated group during the third trimester of pregnancy. Analyzing CD4^+^CD25^+^ Tregs in the neonatal peripheral blood within 6 h postpartum by flow cytometric techniques, they found that the frequency of CD4^+^CD25^+^ T cells in the LdT-treated group was the lowest in contrast to the other categories and highest in nontreated group (LdT-treated group vs. nontreated group vs. HbeAg-negative group: 2.8% vs. 7.0% vs. 4.2%, *p* < 0.05). This phenomenon proved that the ability of CD4^+^CD25^+^ Tregs to stimulate virus multiplication and infant CD4^+^ T cell and CD8^+^ T-cell ratios may be affected by the maternal HBV reproductive status. In addition, the enhancement of neonatal cellular immune function appeared in the pregnant women who were being treated with LDT treatment (87).

#### 5.2.2 Fetal immune and hepatitis B vaccine

The purpose of infant injection hepatitis B vaccine is to boost immunity, resulting in loss of HBsAg and long-term control of HBV replication. According to current research, the recipient’s response to the hepatitis B vaccine may be influenced by their T-cell immunity. For instance, the edited T-cell receptor-chain variable was linked to the immune response of healthy vaccinees to recombinant hepatitis B surface antigen (rHBsAg) as well as the production of hepatitis B surface antibody (HbsAb) (88). Circulating follicular helper T cells (cTfh) and subsets were shown to be involved in the immune response to hepatitis B vaccine injection *in vitro*, and the CXCR3-Tfh cell subset was preferentially activated when HBsAg stimulated PBMCs. One of the reasons for reduced antibody responses in the immune response to hepatitis B vaccination could be a decrease in cTfh cells and subset skewing (89, 90). Approximately 5%–10% of healthy recipients of the hepatitis B vaccine did not produce HbsAb; nonresponse was associated with other factors such as age, gender, body mass, and route of injection, in addition to impaired Th cell response (91).

The HBsAb of those who were effective in the hepatitis B vaccine usually lasted a long time. A study of infants born to HBsAg- and HbeAg-positive mothers found that with or without HBIG inoculation, the fetal HbsAb and immune memory were still persistent 20 years later. Furthermore, if the infants received an interim booster dose of hepatitis B vaccine at age 5, compared with those in the unboosted group, the rate of HBsAb concentration ≥ 10 mIU/ml and the mean HBsAb concentration were higher in the boosted group at the age of 20 (92). In addition, maternal supplementation, such as folic acid, can improve the persistence of protective antibodies; this improvement was linked to the fact that folate deficiency may reduce iron absorption, resulting in anemia, and iron deficiency anemia could influence cell-mediated and nonspecific immunity. Moreover, folic acid may also affect the persistence of HBsAb by providing methyl groups (93). Another interesting question is whether maternal antiviral drugs or HBV markers influence infant immunity response to the hepatitis B vaccine. In order to investigate whether telbivudine exposure during pregnancy influences offspring’s immune response to the hepatitis B vaccine, the researchers enrolled HBsAg-positive mothers and their neonates. All infants had completed a three-dose vaccine schedule at 0, 1, and 6 months. Anti-HBs, immune cells, and cytokines were detected when they were followed up on 11–13 months after birth. The results showed that there was no significant difference between the LdT group and control group in infant anti-HB GMC, positive rate of anti-HBs, strong positive rate of anti-HBs, proportion of myeloid dendritic cells, B cells, helper T cells, cytotoxic T cells, and plasmacytoid dendritic cells, and the concentrations of TNF-α, IFN-α, IFN-γ, IL-2, IL-4, IL-6, IL-10, and IL-12; hence, LdT exposed *in utero* could affect infant immune response to hepatitis B vaccine (94). There was a point where, regardless of dosage, infants were born to HBsAg-positive mothers, and their immune response to the hepatitis B vaccine would not be affected by maternal anti-HB serostatus in the case of successful HBV MTCT. However, HBeAg state had an effect on vaccine efficacy in the lower 5 μg dose; as a result, the infant born to an HBsAg-positive and HBeAg-positive mother had a lower estimated vaccine efficacy value than those born to an HBsAg-positive and HBeAg-negative mother; thus, it was suggested that a higher-dose (10 μg) hepatitis B vaccine was used to prevent HBV MTCT, especially for the infant born to an HBsAg-positive and HBeAg-positive mother (95). Some scholars advised that mothers of infants born HBsAg-positive with HBV DNA loads > 6 log_10_ IU/ml inject 20 μg hepatitis B vaccine, and that increasing the dose of hepatitis B vaccine could protect this high-risk population from occult HBV infection (OCI) (96). A hepatitis B intervention study from Qidong, China, showed that adults benefited from neonatal hepatitis B vaccination, which could protect them from OCI (97). However, a 20-μg hepatitis B vaccine did not reduce the rate of immunoprophylaxis failure compared to a routine dose (10 μg) of the hepatitis B vaccine (96, 98).

#### 5.2.3 Fetal immune and HBIG

HBIG is used to prevent the invasion and replication of HBV, and the recipient can quickly develop passive protective immunity, neutralize and clear the HBV, and avoid HBV infection (99). Compared with the hepatitis B vaccine alone, the immunization schedule of the hepatitis B vaccine combined with HBIG improved the success rate of preventing HBV MTCT (100), and the effect was more pronounced among infants born to HBeAg-positive and HBsAg-positive mothers (101). However, this effect may not be evident in infants born to HBsAg-positive but HBeAg-negative mothers, and some researchers argue that HBIG for an infant who was born to an HBsAg-positive and HBeAg-negative mother was unnecessary for preventing HBV MTCT (102–104). In our opinion, this phenomenon may be related to the low HBV transmission rates between HBeAg-negative and HBsAg-positive mothers and children, but HBIG did not play a role in them. In addition, a number of studies concluded that administering HBIG injections to infants who were born to mothers who were HBsAg-positive but HBeAg-negative did not appear to lower the rate of HBV-MTCT, but it may be helpful in preventing infantile fulminant hepatitis (8, 105). Therefore, evidence that children born to HBeAg-negative and HBsAg-positive mothers are free of HBIG is not sufficient. In addition, in both high and low HBV endemic areas, considering the cost and economic efficiency of vaccination, HBIG treatment for infants whose mothers had HBsAg positivity is a cost-effective addition to universal hepatitis B vaccination, regardless of whether their mothers had HBeAg positivity or not (106–108). Compared to a higher dosage (200 IU) of HBIG, 100 IU HBIG may be enough to block HBV MTCT (109).

## 6 Conclusion and perspective

**Figure 2:**
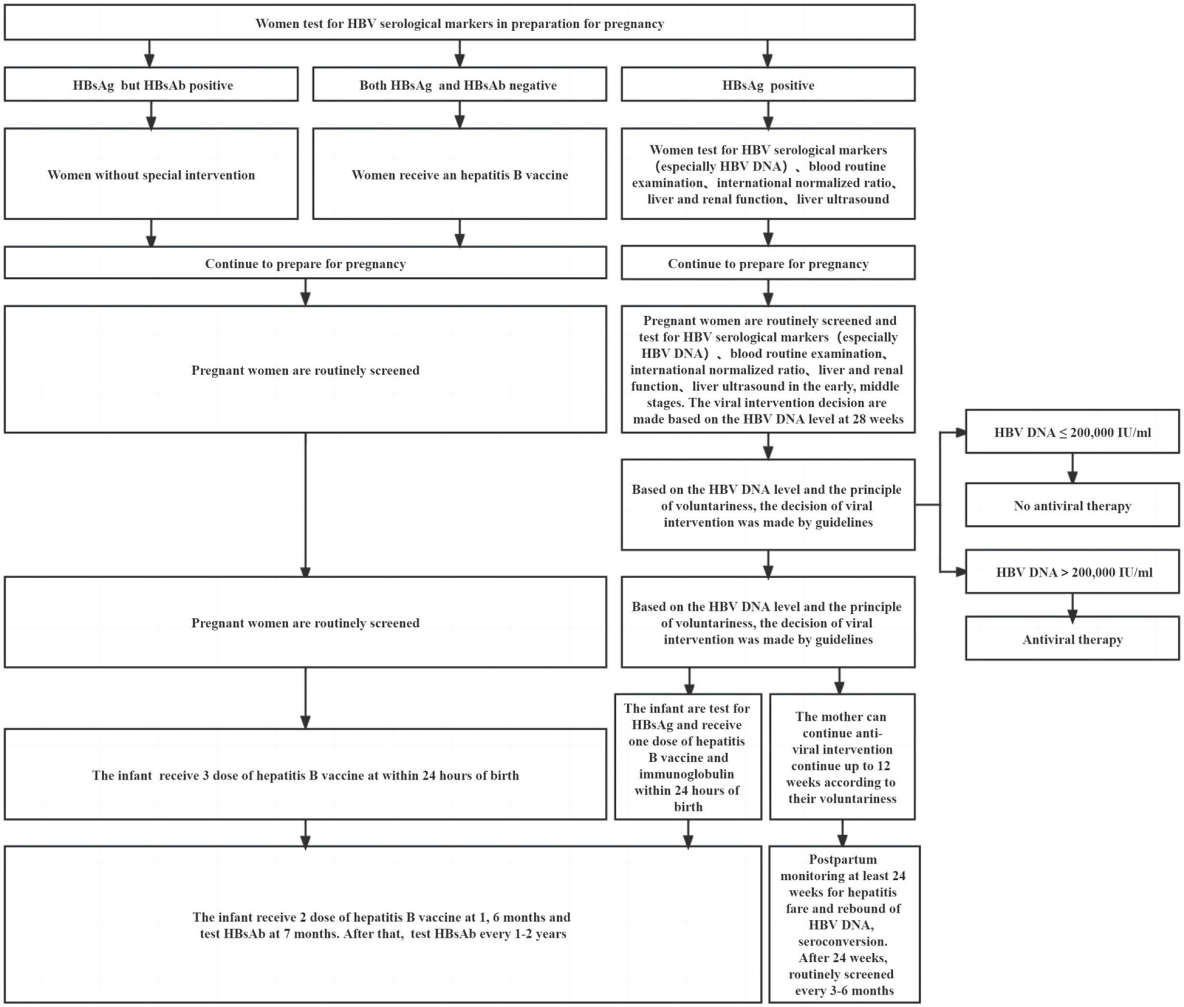
Recommendations for a woman of reproductive age during preparation for pregnancy, pregnancy, and postpartum.

HBV infection can affect human immunity, and the immune environment also changes after pregnancy. Therefore, women who are pregnant and have a chronic HBV infection appear to be at a higher risk of complications during pregnancy compared to women who do not have HBV infection. When maternal immunity is balanced, complications may not occur. Precision medicine in resource-allowable settings requires close monitoring of the mother during pregnancy and postpartum. In addition, people at high risk of HBV transmission can be identified earlier, and intervention to prevent HBV MTCT can begin as soon as possible. Postpartum complications such as ALT flare may not be all bad. Immune-mediated ALT flare may be accompanied by clearance of HBV and may rarely cause hepatic decompensation or even death under close monitoring (31, 110). Hence, chronic HBV pregnant women who use antiviral drugs correctly when postpartum hepatitis occurs and their specific immune environment may favor a good outcome from chronic HBV infection. Some studies indicated that approximately 12%–55% of pregnant women who were HBeAg and HBsAg positive achieved spontaneous HBeAg seroconversion after delivery, which may be related to the presence of mutations in the pre-core, the basal core promoter, or both of those locations combined (111, 112). Timely and appropriate antiviral intervention and standardized vaccination of hepatitis B vaccine and HBIG are particularly important for children born to HBsAg-positive mothers. The recommendations for a woman of reproductive age during preparation for pregnancy, pregnancy, and postpartum care are shown in **Figure 2**. An infant’s immune function is not mature, making them susceptible to certain diseases, slow to recover from diseases, and prone to sequelae. Compared with those infected by horizontal transmission, adult chronic HBV patients with MTCT were susceptible to severe liver diseases and had a poor therapeutic effect (110). In addition to liver cirrhosis and HCC, people with chronic HBV infection also had a higher risk of developing non-Hodgkin lymphoma, pancreatic cancer, and other nonhepatocellular carcinoma malignancies (113, 114). Prenatal HBV exposure alters the epigenome profile (such as DNA methylation) in infants, and the differences in DNA methylation have been linked to the development of hepatocellular and colorectal carcinoma, as well as fatty acid oxidation (115). There are many things we do not know about HBV infection. It is clear that a combining scheme of screening positive mothers, administering antiviral therapy in a high-risk group (HBV DNA > 2 × 10^5^ IU/ml, rather than HBeAg positive or negative) during pregnancy, and implementing infant immunization schedule for administering the hepatitis B vaccine in conjunction with HBIG are required to prevent HBV MTCT. For the sake of postpartum complications and occult HBV infection in children, it is recommended to extend their follow-up time after birth. It is not appropriate to conduct double-blind, randomized trials on pregnant women or infants.

In order to prevent HBV MTCT more successfully, we should conduct feasibility prospective studies, relying on good data retrospectively and immunoassay *in vitro*. For example, observational studies with high confidence are needed to determine whether HBV infection increases maternal and fetal adverse outcomes. In addition, relevant studies of maternal serology are required to prove whether maternal DNA is associated with the failure of HBV blockade. More immune tests and placental pathology *in vitro* should be carried out to clarify the mechanism of HBV MTCT.

## Author contributions

HL, ML, and YX contributed to the study concept and design. HL,WC, LuxZ, LY, XB, YLin, WD, TJ, FS, ZZ, YLu, LuZ, RL, YG, SW, HH, XC, LH, MX, QX, JD, and RS collected and sorted out literature. HL wrote the first manuscript and drew pictures. WC, LuxueZ, LY, XB, and YLin edited and modified the English manuscript. JD, RS, ML, and YX submitted the modified version. All authors contributed to the article and approved the submitted version.
